# Microbial Experimental Evolution – a proving ground for evolutionary theory and a tool for discovery

**DOI:** 10.15252/embr.201846992

**Published:** 2019-07-24

**Authors:** Michael J McDonald

**Affiliations:** ^1^ School of Biological Sciences Monash University Melbourne Vic. Australia

**Keywords:** adaptation, directed evolution, experimental evolution, microbiome evolution, selection, Evolution, Microbiology, Virology & Host Pathogen Interaction

## Abstract

Microbial experimental evolution uses controlled laboratory populations to study the mechanisms of evolution. The molecular analysis of evolved populations enables empirical tests that can confirm the predictions of evolutionary theory, but can also lead to surprising discoveries. As with other fields in the life sciences, microbial experimental evolution has become a tool, deployed as part of the suite of techniques available to the molecular biologist. Here, I provide a review of the general findings of microbial experimental evolution, especially those relevant to molecular microbiologists that are new to the field. I also relate these results to design considerations for an evolution experiment and suggest future directions for those working at the intersection of experimental evolution and molecular biology.

**Glossary**
clonal interference: slowed rates of fixation in an asexual population due to competition between lineages that each carry a beneficial mutation
coverage: the length of concatenated DNA‐sequence read data divided by genome length
*de novo* mutation: a mutation that occurs spontaneously during a period of evolution
fixed: the state at which an allele for a given genetic locus is at a frequency of 1 in a population
genetic barcode: a short DNA sequence that is used to identify an individual or lineage
haplotype: the set of genetic variants physically linked on a single chromosome
HGT: horizontal gene transfer
lineage: a set of individuals that share a common ancestor within a given time period
LN: natural log
LTEE: long‐term evolution experiment
N: population size
parallel evolution: the evolution of similar phenotypes and genotypes in independently evolving populations
selection coefficient(s): a quantitative representation of relative fitness or reproductive success
standing genetic variation: genetic variation that is present in a natural or laboratory population before the period of evolution considered by the observer

## Introduction

**Figure 1:**
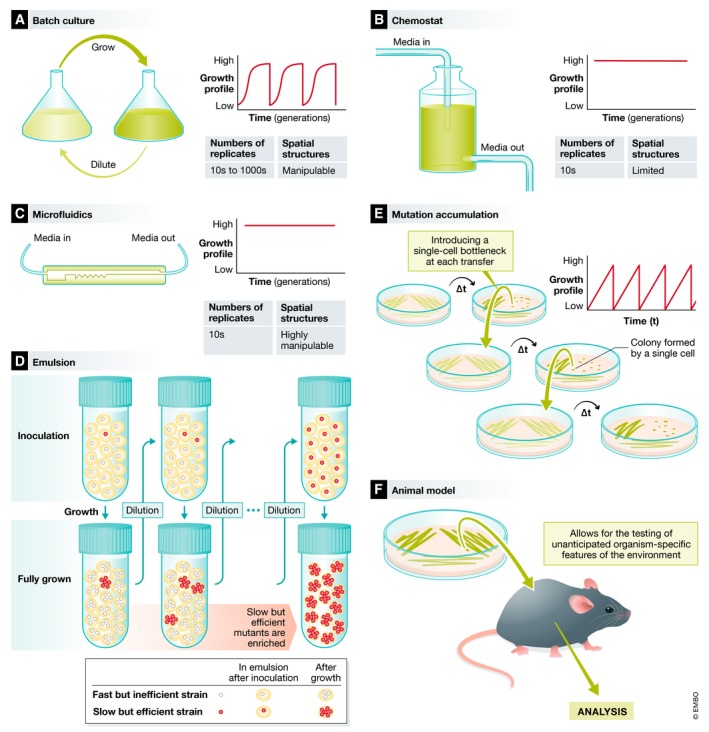
Mechanisms of propagation for experimental evolution (A) Batch culture requires the regular dilution of culture into fresh media. These experiments are relatively easy to establish, since a range of vessels commonly used in a microbiology laboratory can be used for batch culture. These experiments can be scaled to a large number of replicates, for example when using 96‐well plates. (B) Chemostat culture systems include mechanisms for the constant supply of fresh medium. This provides for the continuous cultures of populations and constant growth without large fluctuations in populations size or growth phase. (C) Microfluidics provides the most precise control over the supply of media and supplements to cell cultures. Microfluidics may need to be custom designed, and the number of replicates will be limited. (D) Emulsion cultures take advantage of small cell‐containing vesicles that form when mixing an oil, surfactant and cells. The number of cells in each vesicle is determined by the ratio cell, surfactant and oil. The cells can be mixed back into a single population by vortexing and centrifuging the solution. One advantage of evolving cells in a large number of small populations is that this can select for yield per‐vesicle rather than rapid growth 144. (E) Mutation accumulation introduces a regular, single‐cell bottleneck into each replicate population. This achieved by streaking out cells on a petri dish and then choosing a single colony (founded by a single cell) to streak out the next plate. (F) Microbial cultures can be introduced into a model organism, often a plant or a mouse, and left to propagate for a number of generations before it is recovered from the organism. The recovered cells can be analysed or subjected to further propagation in the organism. This mode of experimental evolution allows for the testing of unanticipated organism‐specific features of the environment that are difficult to replicate in the laboratory.

Experimental studies of evolving populations now constitute one of the foundations of the theory of evolution 1. In particular, studies of microbial populations in the laboratory bring greater power and precision to experimental evolution studies, providing a means to carry out elaborate tests of theory and explore new ideas in evolutionary biology 2. A typical microbial evolution experiment starts with a culture, just like any other in a microbiology laboratory. Cells are inoculated into media and left to grow until the culture reaches a high population density. Instead of throwing out or using all of the resultant population, the experimental evolutionist transfers or dilutes the culture to allow continued growth and division. This cycle can be continued indefinitely, and as the generations accumulate, natural selection will drive the population to adapt to the laboratory environment. This simple process can be carried out in the laboratory using a range of experimental systems, summarised in Fig 1.

### Long‐ and short‐term experimental approaches to study evolution

Perhaps the most striking advantage of experiments with microbes is the access to long evolutionary time scales. The short generation times of microbes allow for up to tens of generations of evolution to pass every day. In theory, an evolution experiment is limited only by how long the experimentalist can maintain regular transfers. A microbial population is easily stored in the freezer, for an indefinite period, so populations can be saved as a frozen snapshot of evolution or used to restart the experiment when inevitable accidents happen. The longest running, and probably most famous, microbial evolution experiment is the long‐term evolution experiment (LTEE). This experiment is comprised of 12 replicate populations of *E. coli*, started in 1987 3 and still passaged daily over 68,000 generations later (see here for a recent review of this experiment 4).

What can be learned from running an evolution experiment for so long? Twenty years ago, an evolutionary biologist might have predicted that these populations of *E. coli* would have reached optimal fitness after a few thousand generations. However, we now know that each population continues to adapt after 61,500 generations 5, 6. A key discovery has been the evolution of the utilisation of citrate (cit+ phenotype), a carbon source used as a buffer in the growth media. The evolution of this phenotype is especially significant because a species‐defining characteristic of *E. coli* is that citrate is unable to be utilised under oxidising conditions 7. The effect of mutations that explicitly cause the cit+ phenotype is dependent on other “potentiating” mutations that do not seem to directly influence citrate utilisation and occurred within the first 20,000 generations of the experiment 8. In other words, this particular trait is unlikely to have evolved in a short‐term experiment.

However, there are quicker routes to study many generations of evolution. An alternative to propagating a few experimental replicates for the long term is to evolve many replicate populations for a shorter period of time. As long as selection is strong, populations can adapt rapidly. Adapting *E. coli* to high temperatures, Tenaillon *et al* propagated 115 experimental populations for 2,000 generations 9. Increasing the number of replicates by another magnitude, Lang *et al* evolved 1,000 replicate populations of *Saccharomyces cerevisiae* for 1,000 generations 10, 11. The massive replication of these studies confers the statistical power to detect evolutionary change, which may be more difficult to detect after only hundreds of generations of evolution. The discovery of the cit+ phenotype shows that there are some questions these highly replicated short‐term studies cannot address; however, there are trends emerging that are consistent across both long‐ and short‐term experiments 12, 13, reviewed below.

### Repeatability, diminishing returns and rapid diversification: predictable trends in experimental evolution

**Figure 2:**
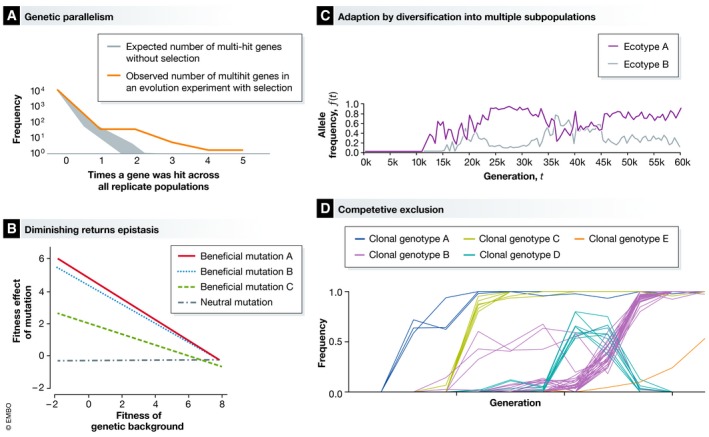
Three consistent results from evolution experiments (A) Genetic parallelism. A signature of natural selection is the repeated evolution of mutations in the same genes in independent populations. The expected number of multi‐hit genes mutated across six replicate populations in a hypothetical 1000‐generation experiment without natural selection (grey shaded) and an example of the number of multi‐hit genes in a population with selection (orange line) 6. (B) Diminishing returns epistasis. The fitness effect of a beneficial mutation is negatively correlated with the fitness of the genetic background in which it occurs (figure adapted from 25). (C) Stable polymorphism can evolve, whereby multiple ecotypes, each adapted to a different niche in the microcosm, coexist in the population. Figure adapted from 27. One possible outcome of experimental evolution is that populations will adapt by successive sweeps of beneficial mutation, occasionally hampered by clonal interference (D).

Parallel evolution is the evolution of the same phenotypes, and sometimes the same genetic mutations, in independently evolving populations 14. Parallelism is often driven by natural selection and has been observed in both short‐ and long‐term experiments across a range of species 11, 15, 16, 17, 18, 19 (Fig 2A). Repeatability in evolution experiments is interesting because it suggests that the phenotypic outcomes of evolution could be predictable. To anticipate the evolutionary response to environmental changes is a major goal of evolutionary biology 20, and the capacity to make accurate predictions of the outcomes of evolution would be desirable. However, it is unclear whether predictions about evolution could ever be precise enough to be useful, and this is subject to ongoing studies using microbial models 21, 22.

At the onset of an evolution experiment, adaptation tends to be rapid and then slows down over time 5, 23. In the *E. coli* LTEE, the rate of fitness increase follows a power law, which suggests that there is no optimal fitness that can be attained by the evolving population 5. The slowed rate of adaptation over time can be explained by epistatic interactions that cause the fitness effects of beneficial mutations to be lower in a better adapted population 24. Experiments show that beneficial mutations engineered into a low‐fitness genetic background have a larger effect than if they are engineered into a high‐fitness background (Fig 2B). This “diminishing returns” epistasis has been observed using beneficial mutations from the experimental evolution of *M. extorquens* and *S. cerevisiae* 25, as well as from the *E. coli* LTEE 26. While diminishing return epistasis makes no predictions about specific phenotypic outcomes of evolution, it does allow for robust predictions to be made about the ongoing rate of adaptation in a population, although this might not be true in populations experiencing fluctuating or complex environments.

Most evolution experiments use unicellular organisms adapting to defined‐nutrient environments. One of the more surprising findings in evolution experiments has been the capacity for these simple experimental systems to evolve diverse, co‐existing subpopulations adapted to different niches, evident in both short‐ and long‐term studies of evolution 6, 27, 28 (Fig 2C). Diverse subpopulations can evolve in response to environmental heterogeneity introduced by the experimenter, or due to a process called eco‐evolutionary feedback 29. As evolution happens in microbial populations, the altered production of waste products or rates of consumption can cause modifications to the environment. This change in ecology alters the selective pressures experienced by individuals and can drive further evolution 30. The observation of eco‐evolutionary feedback in evolution experiments emphasises its importance in real microbial communities and suggests one mechanism that could drive the continuous evolution observed in long‐term evolution experiments.

### Experiments with microbes facilitate precise control over fundamental parameters of evolution: environment, population size and mutation

Understanding, and manipulating, evolution in natural populations is difficult due to the large number of factors that can influence the outcomes of evolution. A major benefit of working with laboratory populations of microbes is the control that can be exerted over the key parameters of evolution: the environment, population size, mutation rate and founding genotype 2. The environment determines the selective pressures experienced by an evolving population and therefore drives the genetic and phenotypic outcomes of evolution. The capacity to maintain many controlled experimental replicates while manipulating a single variable makes possible the observation of potentially subtle effects. The fundamental importance of the environment for interpreting and setting up evolution experiments is discussed below.

Population size (*N*) determines the strength of the selective forces experienced by the population. The minimum effect size of a mutation that can be detected by natural selection, expressed as a selection coefficient (s), is 1/*N*, where “*N*” is the population size, so that selection is ineffective when *N*s < 1 31. A small population is more likely to experience fluctuations in allele frequency due to genetic drift, the random sampling of allele frequencies across generations. This can lead to the chance fixation of deleterious mutations or loss of beneficial mutations. As a consequence of genetic drift, small populations can expect slower rates of adaptation and, in extreme cases, population extinction 32. Some experiments are designed to explore the consequences of variation in population size 33, 34, 35, 36, and may deliberately bottleneck the population to 1–10 individuals (Fig 1). If the goal of the experiment is to avoid genetic drift, a dilution rate that does not bottleneck the population to < 10^3^–10^4^ individuals is recommended.

Variation in the mutation rate allows the experimenter to vary how much genetic variation, the “fuel” of evolution, is supplied to the population 37. The rate of evolution is proportional to the amount of genetic variation in the population 38. While some evolution experiments start with large amounts of standing variation 39, 40, 41, many experiments are founded by a genetic clone 3, 28, 42 and adaptive evolution must therefore be fuelled by *de novo* mutations. In some experiments 43, 44, elevated mutation rates are artificially induced by supplementing growth media with a mutagen or by deleting genes required for mismatch repair.

### Experimental evolution of antimicrobial resistance

The evolution of antibiotic resistance is a global health challenge that, like experimental evolution, sits at the intersection of the disciplines of evolutionary biology, microbiology, molecular biology and genomics 20. Evolution experiments can be used to measure the fitness costs of the mutations that underlie antibiotic resistance 45, 46, 47, 48, 49, and the rate and probability of the evolution of resistance 49. Mutations that cause antibiotic resistance often occur in genes for important biological functions and are therefore expected to cause a reduction in growth rate or viability 50. Fitness assays (see Box 1: How to measure fitness) have shown that the effects of the mutations that confer antibiotic resistance are actually highly variable 47, 51, 52, 53, and do not always come at a cost. When resistance mutations are costly, a resistant microbe can adapt by secondary mutations that compensate for the effects of primary resistance mutations 54.

Since antibiotic resistance is a consequence of evolutionary processes, strategies for the amelioration of antibiotic resistance, especially resistance to multiple drugs, should take evolution into account 55. One promising line of research is to characterise the fitness effects and antibiotic susceptibilities of multidrug‐resistant strains. In order to attain resistance to multiple drugs, multidrug‐resistant strains are likely to have evolved multiple primary resistance mutations as well as several compensatory mutations 54. It is possible that some multidrug‐resistant strains will be less able to evolve resistance to an additional antibiotic 55. Knowledge of the susceptibilities that evolve with multidrug resistance could facilitate the targeted use of drug combinations based on the genotype of clinical pathogenic strains.

**Box 1: How to measure fitness**

Fitness is a quantitative measure of the capacity of an organism to contribute offspring to the next generation. Fitness assays are carried out to determine the degree of adaptation of a population after experimental evolution and to validate the fitness effect of specific mutations. Fitness can be experimentally determined by a wide range of assays. Growth rates 145, total carrying capacity, biomass 105 and speed of colony boundary expansion 141 have all been used as measures of fitness in evolution experiments.

The gold standard for fitness measurement in the laboratory is competitive fitness assays. The starting point for a fitness assay is to obtain or construct a marked reference strain. This is typically the ancestor of the evolution experiment, modified to be readily distinguished from the evolved strain. The nature of the genetic marker can influence the accuracy of the experiment. For instance, if a fluorescent marker is used to differentiate the ancestor from an evolved strain, the proportions of each genotype can be measured using flow cytometry 10 and 10s of thousands of cells can be counted in order to measure ratios. Alternatively, the mixture can be spread onto agar plates containing supplements that provide for the distinction of genotypes 146, and this allows for the counting of hundreds of cells. Initially, each strain to be measured should be mixed with the marked reference strain in a 1:1 ratio. Even if care has been taken to mix the competitors in a 1:1 ratio, it is very important to measure the initial starting frequency, since small difference in this ratio can have a large effect on the calculations for fitness.

Once a portion of the mixture has been taken aside to measure the starting ratio, the mixture of competing cells is diluted and incubated for a set period of time, allowing for the two genotypes to compete. After this period of competition, the proportions of the genotypes are measured again. The selection coefficient can be calculated from these two measurements by counting number of evolved individuals and dividing by the number of reference individuals. This is done for the initial time point and the final time point. The final ratio is divided by initial ratio, and the natural log (LN) of the quotient gives a measure of the performance of the evolved strain, compared with the reference strain. This value is divided by the number of generations that passed between the final and initial time points, yielding a per‐generation selection coefficient (s). The period of competition between these measurements must be chosen carefully. If left too long, then one genotype will drive the other to extinction, thus reducing the precision of the calculation of s. If the competition is too short, then the genotype frequencies will not have changed enough to allow the detection of differences between the genotypes.

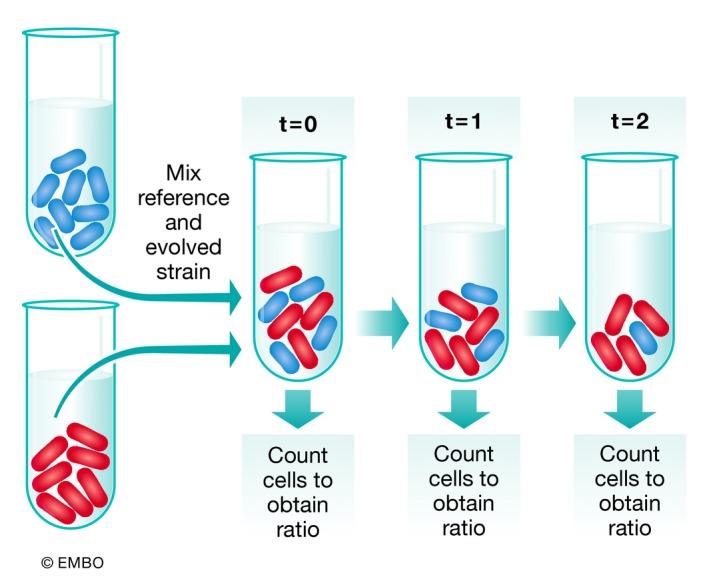

### Experimental evolution of bacteriophages

Bacteriophages are another source of antimicrobial therapies 56, and experiments with bacteriophage provided some of the first insights into the genetics of adaptation in evolving laboratory populations 57. Bacteriophage genomes are small, and whole‐genome sequencing of experimental phage populations was possible before the rise of next‐generation sequencing technologies 58. This head start was exploited to identify the mutations that evolved in phage evolution experiments and to measure the fitness effects and epistatic interactions of these mutations 19, 59, 60. The relative ease of propagating bacteriophage and bacteria in co‐culture has also led to insights into co‐evolutionary dynamics. For instance, bacteria that are propagated with an infecting phage experience more rapid molecular evolution than bacteria evolved in isolation 61, 62. If the bacteria are propagated with diverse bacteriophage, the rate of evolution increases with the number of phage types present in the culture 63.

Bacteriophages typically bind to a membrane protein to gain entry to the cell. Experimental evolution has facilitated the detailed molecular analysis of the mechanism by which phage λ can evolve to bind a new site on the *E. coli* membrane 64, 65. Conversely, bacterial resistance to a bacteriophage can evolve by modifying or deleting the gene that encodes that protein. Since antibiotic resistance is sometimes conferred by multidrug efflux pumps, it has been hypothesised that a phage that targets such a pump could be given in tandem with the antibiotic and thus comprise an “evolution proof” treatment strategy 56. This principle was demonstrated with a phage selection experiment, which drove the selective loss of the MEX efflux pump, thereby restoring antibiotic sensitivity to a multidrug‐resistant strain of *P. aeruginosa* 66.

### Experimental evolution as a tool

Microbial evolution experiments are often designed with the goal of providing insights into evolutionary theory, or the evolution of a particular trait. However, experimental evolution is also a tool that can be used to evolve organisms for specific applications. The introduction of genetic modifications designed to confer useful properties often results in slowed growth or other reductions in performance 67. In yeast, engineered strains can be crossed with “wild‐type” strains and then passaged to promote recombinants that possess the engineered features as well as the productivity of fast‐growing strains 68. Continuous passaging has been used widely to restore growth rates in *S. cerevisiae* engineered for the production of ethanol and the consumption of xylose 69, 70, 71. In a recent example, the engineered *E. coli* strain C321 has been modified to replace all UAG codons with UAA. This strain provides the ideal genetic background for a range of biotechnological applications, such as the incorporation of codons for non‐standard amino acids into the genetic code. However, the engineering of this strain caused slow growth. A 1,000‐generation evolution experiment propagating this strain in the laboratory resulted in the evolution of restored high growth rates. Moreover, whole‐genome re‐sequencing of the evolved populations revealed the mutational targets of selection and therefore the causes of reduced growth rate in the founder strain 72.

Experimental evolution can be used to adapt microbes to novel hosts, as well as novel laboratory conditions. Some species of *Wolbachia* bacteria spread quickly among their hosts by conferring a reproductive advantage to infected females. In addition, some *Wolbachia* strains are able to induce resistance to insect pathogens. A strategy was devised to spread resistance to the Dengue virus amongst mosquitos using a strain of *Wolbachia* originally discovered in *D. melanogaster*. However, this strain was not suited to rapid growth and dispersal in mosquito populations. In order to adapt this *Wolbachia* strain to grow in the *A. aegypti* mosquito's intracellular environment, it was passaged in a mosquito cell line for 2 years. After this period, the newly evolved *Wolbachia* strain was able to establish a stable infection in mosquitos 73 and thereafter facilitate the eventual public dispersal of dengue resistance mosquitos in Australia 74.

Next, I introduce important considerations for the design of evolution experiments by describing some key results in experimental evolution. This review does not provide a full historical treatment of experimental evolution. I recommend these books 1, 2 and these reviews 75, 76, 77 for exhaustive treatments of earlier periods and non‐microbial experimental evolution, and these reviews 78, 79, 80, 81 for different aspects of experimental evolution.

## You get what you select for: environment and the outcomes of experimental evolution

One of the most important choices when starting a laboratory evolution experiment is the environment. Setting conditions beyond what is normally experienced by an organism will drive adaptation. Adaptation to a range of conditions has been described, including elevated temperatures 9, 82, antibiotic gradients 55 and even high levels of ionising radiation 83. The environmental parameters that can be used to drive selection are limited only by the imagination. As long as the chosen environmental parameter provides a selection pressure that drives the differential survival of individuals in the population, adaptation will happen. While experimental populations can be relied upon to adapt regardless of the selective pressure, the types of adaptations that evolve can be difficult to predict. Wildenberg *et al* 84 used a fluorescence‐activated cell sorter to select for the brightest fluorescent *S. cerevisiae* cells every 24 h. One anticipated outcome was that selection would drive the evolution of gene expression in order to modulate fluorescence. Instead, the population evolved to periodically form multicellular clusters that increased brightness and thereby conferred a selective advantage. This unpredicted outcome did not diminish the elegance of this experiment. However, it serves to demonstrate how unpredictability can thwart experiments that are designed to have specific outcomes.

In general, the more complicated the selection regime, or subtle the strength of selection, the more unpredictable the outcomes of evolution. It should be noted that a complicated, but well‐designed, experiment can still elicit the anticipated response to selection. One experiment sought to evolve multicellular traits by selecting for or against germ progenitor cells in cooperative mats of the bacteria *P. fluorescens*. Although the genetic mechanisms of evolution were unexpected, the experiment successfully applied selection pressures that led to the evolution of multicellular traits 85.

### Simple environments can drive the evolution of loss of function

Natural environments expose microorganisms to a range of nutrients and stresses that vary across spatial and temporal scales. The complexity of natural environments is reflected in the large numbers of genes that organisms have evolved to utilise diverse nutrients and respond to stress. Laboratory experimental populations experience environments that are typically much less complex 86, and will adapt by mutations that inactivate genes that have become superfluous in the conditions of the experiment 12, 87.

Many evolution experiments are carried out in growth media that contain a single carbon source, usually glucose. In the LTEE, glucose is supplied as the sole carbon source in a concentration that limits population growth 3. In an evolution experiment, the regular supply of glucose every 24 h can lead to the evolution of a reduction in the “lag time”, the time required for the population to enter the log growth phase. In the LTEE, this is achieved via mutations in *pykF,* which became fixed in every population within the first 2,000 generations of the experiment 6, 88. Adaptation for specialisation on a single carbon source can come at the cost of growth on other carbon sources. Studies of the LTEE after 2,000 generations of evolution showed that the *rbs* operon, which encodes proteins required for the utilisation of ribose 89, has been disrupted or deleted in all 12 replicate populations. Measurements of the selective benefit of *rbs* loss using competitive fitness assays determined the fitness gain to be ~1% 90. Since then, whole‐genome sequencing has revealed the disruption of other genes, including genes for the utilisation of carbon sources, such as maltose, that may be superfluous in the minimal media environment.

Other evolution experiments have propagated the yeast *S. cerevisiae* in media containing high concentrations of glucose, in a range of culture conditions. Genes that evolve beneficial mutations in multiple replicate populations during adaptation to high glucose concentrations in batch culture have been shown to be targets for selection across different experiments 11, 25, 91. Whole‐genome sequencing of evolved populations has revealed that over half of the replicate populations adapt by mutations that disrupt genes that encode negative regulators of the RAS/PKA pathway 11, 91. These mutations increase RAS/PKA pathway activity and result in the rapid utilisation of glucose, even in experimental cultures carried out in a range of glucose concentrations 86. Mutations in RAS/PKA pathway genes have also been discovered in chemostat experiments with glucose‐limiting concentrations 87. Interestingly, in these experiments the most frequently recovered mutations increase the amount of glucose transport. Since glucose is the limiting nutrient in these experiments, increasing transport of glucose into the cell may be a rapid path to adaptation. However, the frequent recovery of mutations in genes that increase activity of the RAS/PKA pathway in experiments with high and low glucose, and both batch and chemostat culture, suggests that glucose may be the selective force driving the recurrent evolution of these mutations.

Evolution experiments have shown that genes required for functions beyond metabolism are also targets for loss‐of‐function mutations. After 1,500 generations, replicate populations of *M. extorquens* were found to have sustained large deletions of up to 10% of their genome, covering a wide range of gene functions 92. *S. cerevisiae* evolution experiments often employ strains that have been genetically altered to reduce the probability that they can mate. Propagation without sexual reproduction can cause selection for mutations that interrupt the genes that encode components of the mating pathway 11. Careful measurements have shown the fitness gain derived from eliminating the expression of an unnecessary gene. The precise cost of expression of one gene in the mating pathway was determined to be approximately 1% 90, demonstrating that the expression of un‐needed genes is a costly trait that can be targeted by selection in evolution experiments.

The selective benefit of loss‐of‐function mutations drives their fixation 93. However, genetic target size for this class of mutations is another factor that makes these genes more likely to contribute towards adaptation. Any frameshift or change in a key amino acid can result in a non‐functional protein. Gain‐of‐function mutations require modification of certain amino acids that will increase the activity or function of that protein 17. Any given mutation that occurs in a gene is therefore more likely to cause a loss than a gain in function. This trend of evolution by loss of function has been borne out in bacterial evolution studies 12, 17, 89, and studies of haploid *S. cerevisiae* 11, 87, 91. However, the few studies that have studied the evolution of haploid and diploid *S. cerevisiae* in similar environments have found different molecular patterns of adaptation. This may be because the inactivation of one of two gene copies in a diploid is less likely to cause a phenotypic change 94. It should be noted that relatively few mutations that occur in evolution experiments have been characterised and that mutations that occur across multiple experiments are more likely to be studied. The striking difference between molecular adaptation in diploid and haploid *S. cerevisiae* suggests that small differences in experimental conditions can lead to large differences in the outcomes of evolution and that the lessons learned in one experiment should only be tentatively applied to other experimental systems.

### Spatial structure selects for diversification

Many microbiology protocols specify that cultures are well‐shaken and aerated. In experimental populations, this generates a homogenous distribution of nutrients and oxygen and promotes a uniform selection pressure through the microcosm. When cultures are incubated statically, without shaking, new environmental niches become available on surfaces and across nutrient gradients, and the outcomes of evolution can be quite different. In 1,000 experimental populations of *S. cerevisiae*, 10% of replicates were found to have evolved stable, co‐existing subpopulations, one that can attach to the wall of the growth chamber, and another that grows at the bottom 10. Whole‐genome sequencing and genetic reconstructions revealed that this wall‐attachment adaptation was repeatedly conferred by mutations that disrupted ergosterol biosynthesis 27.

The most comprehensive exploration of evolution in static microcosms has been carried out using *P. fluorescens* 17, 28, 95. In an adaptive radiation that reliably unfolds over 7 days, a planktonic ancestor diversifies into genetically distinct lineages. The best studied of these is the “wrinkly spreader”, which adapts by forming a mat of stuck‐together cells that float on the broth surface and attach to the glass walls of the microcosm. This adaptive strategy provides access to oxygen, a limiting nutrient in a non‐shaken broth, and to nutrients in the liquid phase. The mutations that cause the wrinkly spreader phenotype modify expression of a secondary messenger molecule, c‐di‐GMP 96, causing the constitutive expression of cellulose. Even though there are over 25 c‐di‐GMP‐producing enzymes in the *P. fluorescens* genome, only three of these are ever mutated during wrinkly spreader evolution 17. If all three of the operons encoding these enzymes are removed from the genome, then this triple deletion mutant can evolve the wrinkly spreader phenotype by mutations in some of the other genes that encode c‐di‐GMP proteins 17, 97. Parallel evolution is commonly observed in natural and experimental populations, and it has long been hypothesised to be due to the organisation and content of the genome as well as natural selection 14, 98. The molecular genetic analysis of the trait combined with a delete‐and‐evolve strategy provided an early demonstration of the genetic constraints on evolutionary outcomes.

The technique of static incubation in the presence of a surface has been used to explore adaptation to surface attachment in other species, such as the pathogens *B. cenocepacia* 18, *P. aeruginosa* 99, *S. typhimurium* 100 and *V. cholerae* 101. A clever study of attachment and biofilm evolution was carried out by the Cooper laboratory 18, 102. A plastic bead coated with a *B. cenocepacia* biofilm was incubated in media containing another bead. After 24 h of incubation, this second bead was removed and used to found a new culture. This was continued for 143 days (~1,500 generations) 102 and drove the evolution of the rapid and robust biofilm colonisation of the second bead by the inoculating bead. The authors found that three types would reliably evolve, forming a biofilm community of increased productivity relative to a biofilm formed by any one type. Interestingly, the variants that evolved in this experiment also carried causal mutations in c‐di‐GMP‐regulating enzymes, similar to the genetic causes of adaptation in the *P. fluorescens* “wrinkly spreader” experiments described above. This experiment highlights the potential for experimental evolution to advance the understanding of bacterial attachment and biofilm formation, which is associated with pathogenesis and antibiotic resistance in a range of species 103.

Microfluidics provides a system for the growth and continuous propagation of experimental populations 104. Since the flow rate of media can be carefully controlled, cells can attach to surfaces, while allowing for some population turnover and the constant provision of nutrients. Microfluidic systems have been used to demonstrate the increased productivity of a simple engineered community of *P. putida* and *Acinetobacter* sp. 105. Despite interspecies interactions frequently occurring in naturally occurring microbial communities, establishing a stable, long‐term co‐culture can be difficult in batch or chemostat experimental systems. The capacity to engineer and control the space where microbes interact can facilitate co‐culture experiments and has been used to systematically screen the potential for multiple strains of *P. aeruginosa* to co‐establish biofilms 106.

Microfluidic devices have been underutilised in experimental evolution. One reason may be the perception that specialist knowledge is required to design and construct microfluidic systems and to propagate many replicate populations in parallel. Currently, the design of scalable experimental systems is making microfluidics more accessible 107. Attachment can also occur on biological surfaces. The gut‐on‐chip systems developed for culture of mammalian cells can support the propagation of bacteriophage 108. Similar systems could be exploited to study the evolution of microbial colonisation on biological surfaces.

## High‐throughput methods for identifying and tracking beneficial mutations in evolving populations

Whole‐genome re‐sequencing of replicate evolved populations is now a routine part of experimental evolution 109. The gold standard for assembling and analysing whole genome short‐read data from microbial evolution experiments is breseq, a set of tools developed in the Barrick Lab 110. An important consideration before undertaking a genome sequencing experiment is the method of sampling the population. One approach is to sequence individual clones sampled from evolved populations. In experiments where multiple clones from a single population have been sequenced, it has been found that, as well as mutations that have fixed in the population, the clone will contain mutations that are unique to that clone, also called private mutations. The conclusions that one may draw from the sequencing of a single clone from an evolved population are quite limited since it will be impossible to tell which mutations are rare, and which are high‐frequency mutations that are more likely to have contributed to adaptation.

One way to obtain detailed information about the frequency of each mutation within the population is to carry out whole‐population sequencing 11. The lower bound of allele frequencies that can be detected using whole‐population sequencing is determined by the average sequencing depth or coverage. The theoretical minimum frequency that can be detected is the inverse of coverage (*C*) of the genome. For example, an experiment that obtains whole‐population whole‐genome coverage of 100‐fold depth will not be able to detect mutations with a frequency of < 1%. In reality, due to variations in coverage and the risk of false positives, a conservative approach in this case would be to reject as spurious mutations that do not exceed a frequency of 10% in the sequence data 6, 111. The whole‐genome whole‐population sequencing approach has several weaknesses. The first is the inability to determine which mutations are physically linked on chromosomes (haplotypes). This can be resolved by supplementing whole‐population sequencing with the sequencing of clones. It can be also difficult to detect structural rearrangements, large indels and changes in ploidy, although variations in read depth can provide some information for high coverage data. The only way to unambiguously resolve these types of mutations is to incorporate long‐read sequencing data into the genome assembly 112. There are now tools available for combining long‐ and short‐read data to assemble closed genomes 113.

### Identifying and validating beneficial mutations

Before next‐generation sequencing, it was difficult to discover mutational changes in experimentally evolved populations. Now, the challenge is to determine which of the many mutations revealed by sequencing are actually the cause of adaptation, a problem that has also emerged with the sequencing of tumour genomes 114. If multiple populations have been sequenced, the repeated observation of mutations in the same gene (parallel evolution), across independent replicate populations, can indicate the action of natural selection 6, 9, 11, 17, 19. Statistical tests comparing the observed number of parallel mutations to a null model can be used to determine a conservative cut‐off. This null model should take into account gene size, since large genes are more likely to be mutated during an experiment 72. It should be noted that the absence of parallel evolution is not evidence for the absence of natural selection. These strategies can identify candidate beneficial mutations; however, validation requires that the mutation is either engineered into the ancestral genome or replaced by the ancestral sequence in the evolved strain.

While CRISPR‐cas9‐based technologies are making genetic reconstruction realistic for a growing number of experimental models, it is difficult to engineer and measure more than 10s of individual mutations in a single experiment. Experimental systems, such as *S. cerevisiae*, that allow mating or recombination, can overcome this limitation 111, 115. Haploid evolved clones that carry several mutations can be crossed with the haploid ancestor strain of the opposite mating type. The resulting diploid can be sporulated, generating millions of recombinant haploid offspring, with every different combination of the mutations. This pool of mutants is purged of one mating type 115, to prevent mating of the recombinants and then propagated for a period of 100 generations. The population is then sequenced at multiple time points. This technique is dependent on the capacity for recombination, and so can only be adapted to other sexual eukaryotes or potentially, naturally competent bacterial systems.

### Clonal interference and recombination impact evolutionary outcomes

Whole‐population (metagenome) sequencing across different time points of an evolution experiment makes it possible to track the dynamics of individual mutations that arise and segregate in an evolving population 6, 11, 16, 111, 116, 117. Studies that employ this technique provide a direct view of the trajectories of mutations and how they interact during adaptation. A phenomenon that has been explicated by these studies is clonal interference. Clonal subpopulations arise and compete in experimental populations that contain multiple beneficial mutations. If beneficial mutations arise on different genetic backgrounds, and recombination cannot bring them together, these beneficial mutations will compete. Since the result of clonal interference is one beneficial lineage outcompeting another, some beneficial mutations are driven extinct in the population 6, 11. Clonal interference thus slows population adaptation.

The tracking of mutations through time provides an opportunity to study sex and recombination. Sexual recombination or horizontal gene exchange is common in natural populations of microbes 118. However, the direct observation of the recombination of mutations during adaptation in laboratory populations of microbes is difficult. One of the main reasons for this is that many model laboratory organisms, such as *E. coli*,* S. cerevisiae* or *Pseudomonas* species, do not undergo recombination or HGT under commonly used experimental conditions. These challenges can be overcome by engineering strains of *S. cerevisiae* 44, 111, 119, 120, 121 and *E. coli* 122, 123, 124 that are capable of repeated bouts of sex, or conjugative gene exchange, in an experimental evolution setting.

### Sex can accelerate adaptation in laboratory experimental populations

*Saccharomyces cerevisiae*, with two mating types and the capacity to reproduce both sexually and asexually, provides an ideal experimental system for the study of sexual recombination. This has long been recognised, and the effects of recombination on the rate of adaptation have been investigated in some depth 44, 120, 121. A recent study has added to this by providing details of the genetics of adaptation in recombining populations of *S. cerevisiae* 111. In control populations that did not undergo recombination, significantly deleterious mutations were able to fix 111. This is possible because, as long as the cumulative effect of the mutations in any given genetic background has an overall beneficial effect, a strongly beneficial mutation can mask the effect of a deleterious mutation 125. In populations that were able to recombine the genomes of individuals, these deleterious mutations were decoupled from the beneficial mutations and purged from the population 111. Another potential benefit of recombination is the resolution of clonal interference 38, 126. In populations that do not have recombination, different beneficial mutations were unable to be brought together onto the same genetic background. However, sexual populations were able to fix all of the beneficial mutations that were measured 111.

Experimental evolution of HGT in bacteria has been more difficult to study; however, recombination has been incorporated into *E. coli* experiments using conjugation systems 123, 127. One of these studies confirmed results in *S. cerevisiae* that incorporating recombination into an experimental system will speed up adaptation 123. The benefits of recombination depend on the presence of multiple mutations concurrently segregating in the population. Interestingly, an experiment showed that combining high mutation rates with recombination could further increase the rate of adaptation 124.

### Amplicon sequencing facilitates the tracking of a multitude of lineages

**Figure 3:**
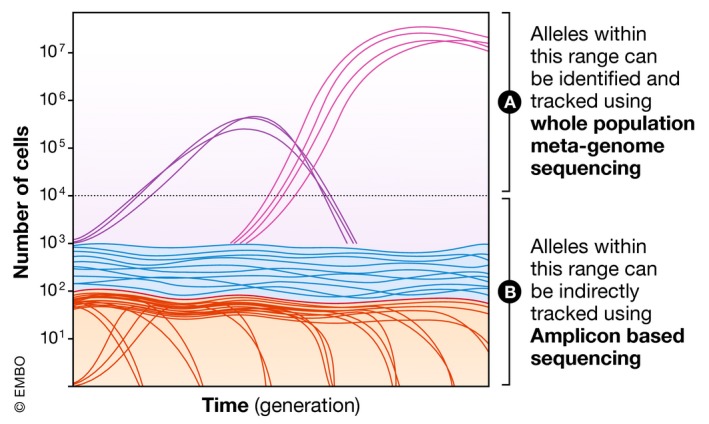
Tracking the dynamics of mutations that underlie adaptation using DNA sequencing Each line shows the trajectory of a mutation that arises during evolution. (A) Whole metagenome sequencing can track all mutations in the genome, but is limited to tracking mutations that attain a high frequency, typically > 1–10%. (B) Amplicon sequencing can track the change in frequency of as many as 500,000 distinct genetic lineages, but does not convey the identity of the beneficial mutations that cause adaptation.

Whole‐population sequencing provides for the detection of any mutations that have occurred across the genome; however, this comes at the cost of sequencing depth. If a population is sequenced to 100‐fold coverage, in theory, only mutations that are present in at least 1% of individuals can be detected (Fig 3). Practically, whole‐population sequencing projects can reliably track mutations that exceed a frequency of 5–10% at multiple time points. This means that within a population of 10^7^ individuals, mutations that attain a sub‐population size of < 100,000 individuals will not be discovered.

One way to circumvent this limit is to sequence a much smaller part of the genome that has been modified to be highly variable (bar‐coded). By sequencing only this portion of the genome to ultra‐high depth (10^5^–10^6^ coverage), many lineages can be tracked. An experiment pioneering this approach has been carried out in a *S. cerevisiae*. Amplicon sequencing at several time points in large populations in batch culture facilitated the tracking of 500,000 individual lineages for 160 generations of evolution 91, 128. Estimations of fitness effects of the mutations that defined each of these lineages revealed the large amounts of beneficial genetic variation that lie hidden in large populations, and provided the most comprehensive picture of the distribution of the fitness effects of new mutations 128 and their dynamics 129. One limit of this technique is that mutations can only be tracked in the short term. As soon as one mutation has fixed in the population, then one version of the barcode also fixes, sweeping out all other barcode variants.

Transposon mutagenesis is a classic technique in bacterial genetics for the functional characterisation of a genome 130. Lineage tracking by amplicon sequencing can be used in combination with transposon mutagenesis to discover novel gene functions 131. Selection is applied to the library by continuously passaging the population in defined growth conditions. By sequencing at different time points, the proportions of all mutations both before and after selection can be compared, and precise fitness coefficients calculated. Alternatively if the selection is applied over longer terms, the presence and absence of certain mutants after selection can be recorded. This technique has been used effectively to systematically identify the genes important for antibiotic resistance in a range of bacteria 132, 133.

## Future outlook

### Experimental evolution of model microbiomes

Experimental evolution of microbes has provided a detailed picture of the molecular details of adaptation. The most comprehensive understanding has come from the simplest evolution models, typically haploid populations of non‐recombining microbes, evolving in isolation from other species. On the other hand, the culture‐free, sequence‐based characterisation of microbiomes has revealed that natural and clinical populations are more likely to evolve as part of a complex community of microbes and to engage in horizontal gene transfer. A new challenge for experimental studies is to understand how evolution happens in these communities and whether the “rules” of evolution discovered so far hold true in these systems. So far, experimental co‐evolution has mainly been explored using bacteria and phage as models of predator–prey interactions 62, 134, 135 or using naturally interacting sets of uncharacterised bacteria 136. There is potential to undertake co‐evolution experiments with well‐characterised prokaryotic and eukaryotic microbes to study the genetic basis of a wider range of co‐evolutionary interactions (see Box 2: In need of answers).

Recent studies have shown that recombination can increase the power of natural selection 111. This insight has been applied in the field of directed evolution, which use recombination to generate diverse combinations of protein domains 137. Incorporating recombination into evolving populations also has the capacity to improve the directed evolution of whole organisms and genomes, not just single proteins. Currently, microbial experimental systems are limited by an inability to readily exchange DNA without cycles of genetic manipulation and induced transformation. However, there are model systems, such as *H. pylori*, where recombination and genetic exchange are constant within the evolving population 138. Such model systems could speed adaptation in directed evolution experiments.

**Box 2: In need of answers**

- **Studies show that antagonistic co‐evolution drives rapid evolution. On the other hand, communities may have a stabilising effect, reducing the impact of environmental change and thereby slowing evolution. How does the rate of adaptation change in microbial communities?** This question can be addressed by co‐culture experiments of model microbial prokaryotic and eukaryotic species with well‐characterised genomes and the capacity for genetic manipulation.
- **Comparative genomics show that HGT is very important for microbial evolution, however experimental tests have been difficult to carry out. How will HGT transform adaptation?** New model systems that more readily exchange DNA in laboratory culture are needed to address this question.
- **Recombination and genome shuffling can speed up adaptation. Can we build systems where adapting organisms of interest can exchange DNA regularly during directed evolution?** This can be achieved by employing naturally competent models, or by developing technologies to make model organisms competent.
- **Since most microbial species exist in communities, what proportion of all genes are required for interactions with other species? Can these explain the large number of genes of unknown function?** Systematic gene deletion collections (with a focus of genes of unknown function) can be co‐propagated within a microbial community to determine whether any genes have a function related to interactions with other species.

### Experimental evolution as a genetic screen for functional annotation of hypothetical genes

Despite being studied intensely for most of the last century, *E. coli* and *S. cerevisiae* still have many genes that have only been assigned hypothetical functions 139, 140. Deletion collections for *E. coli*,* S. cerevisiae* and now other species have revealed the epistatic interactions of many of these genes and provide resources for the connection of gene function with environmental conditions 141. For instance, the systematic plating out of every viable gene mutant on media containing a drug quickly reveals which genes are essential for detoxification or toxicity of the drug. However, some discoveries require the passage of multiple evolutionary generations. One relatively unexplored path towards understanding the importance of these genes could lie in propagating these mutants, or libraries of mutants, in complex conditions. Natural populations of microbes rarely experience a constant environment, yet the default for any experiment with microbes is monocultures growing in constant experimental conditions 76. Moreover, in natural systems almost no species evolves in isolation from other species 142. The last 10 years of microbiome metagenomics has revealed that natural microbial ecosystems are complex 143. Could it be that the genes of unknown function are involved in interspecies interactions? One way to address this is experiments with libraries of deletion mutants in co‐culture with another species. Controlled experiments that compare the performance of deletion mutants in co‐culture and mono‐culture may reveal new gene functions.

Microbes, microbiomes, and the drugs and molecules that they produce emerged from evolutionary processes. Previous progress in molecular biology has relied on converting discoveries such as restriction enzymes, transposons and DNA polymerases into tools to generate new insights. The creative application of experimental evolution to problems in molecular biology has the potential yield the next generation of discoveries into basic and applied biology.

## Conflict of interest

The author declares that he has no conflict of interest.
